# Structural, biochemical, and physiological characterization of photosynthesis in leaf-derived cup-shaped galls on *Litsea acuminata*

**DOI:** 10.1186/s12870-015-0446-0

**Published:** 2015-02-21

**Authors:** Meng-Yuan Huang, Wen-Dar Huang, Hsueh-Mei Chou, Chang-Chang Chen, Pei-Ju Chen, Yung-Ta Chang, Chi-Ming Yang

**Affiliations:** Department of Horticulture and Biotechnology, Chinese Culture University, Taipei, 111 Hsulin Taiwan; Department of Agronomy, National Taiwan University, Taipei, 106 Daan Taiwan; Department of Biotechnology and Pharmaceutical Technology, Yuanpei University of Medical Technology, Hsinchu, 300 Taiwan; Miaoli District Agricultural Research and Extension Station, COA, Guannan, Kungkuan, 363 Miaoli County Taiwan; Department of Life Science, National Taiwan Normal University, Taipei, 116 Wenshan Taiwan; Biodiversity Research Center, Academia Sinica, Taipei, 115 Nankang Taiwan

**Keywords:** Cecidomyiidae, Gall, *Litsea acuminate*, Photosynthesis, Chlorophyll fluorescence, Sink

## Abstract

**Background:**

The source and sink relationships between insect-induced galls and host plant leaves are interesting. In this research, we collected cup-like galls induced by *Bruggmanniella* sp. (Diptera: Cecidomyiidae) on host leaves of *Litsea acuminata* and assessed them to investigate source-sink relationships between galls and host leaves. We characterized several of their photosynthetic characteristics including chlorophyll fluorescence (Fv/Fm), stomatal conductance, and photosynthetic capacity, biochemical components such as total soluble sugar, starches, free amino acids, and soluble proteins. The structural analyses were performed under confocal, light, and scanning electron microscopies.

**Results:**

Compared with host leaves, galls exhibited slightly lower chlorophyll fluorescence; however, stomatal conductance and photosynthetic capacity were not detected at all. Galls accumulated higher total soluble sugars and free amino acids but less soluble proteins than host leaves. No stomata was observed on exterior or interior gall surfaces under light or scanning electron microscopy, but their inner surfaces were covered with fungal hyphae. Confocal imagery showed a gradient of chloroplasts distribution between gall outer and inner surfaces.

**Conclusions:**

Our results strongly suggest that leaf-derived cecidomyiid galls are a type of chlorophyll-deficient non-leaf green tissue and consists on a novel sink in *L. acuminate.*

## Background

Insect larvae residing inside galls use these leaf-derived structures as shelters for protection and sources of nutrition. More than 65% of galls, with various appearances and colors, are derived from the leaves of their host plants within which the larvae reside. Three major hypotheses involving nutrition, environment, and enemies have been postulated to explain the adaptive significance of gall induction and understand the evolution of gall morphology [1]. However, the source-sink relationships between insect-induced galls and host leaves are still disputed. Gall-inducing insects have developed highly specialized and nutritional relationships with their host plants because these insects spend major portions of their lives within galls. They interact with galls by the simple removal of tissue or by damaging vascular tissues in order to manipulate the synthesis and transport of host plant nutrients [2-5]. Also, plants can use the galls as sinks for nutrients for insects’ growth and reproduction [6,7].

We have previously pointed out that prior studies on gall-caused impacts to host leaf photosynthesis do not suggest any general trends; however, Yang et al. [8,9] reported a range of effects from negative to positive. This lack of pattern has not been confirmed within the past decade, therefore this question still remains under dispute and requires further exploration.

Regardless of whether net photosynthesis is directly measured in galls or estimated from radioactive labeling experiments, the photosynthetic rates in galls are usually much lower than in unattacked normal leaf tissues [10]. Aldea et al. [11] found lower photosystem (PS) II efficiency, as determined by chlorophyll fluorescence (Fv/Fm), in *Cecidomyia* galls on *Carya glabra* leaves, *Cynipid* galls on *Quercus velutina* leaves, and *eriophyid* galls on *Ulmus alata* leaves compared to uninfected leaf surfaces. Photosynthetic rates of galled leaves, measured by gas exchange, were reduced when compared to ungalled leaves of naturally growing *Prunus serotina* and *Rhus glabra* [12]. Water potential, photosynthesis rate (indicated by gas exchange), transpiration, and stomatal conductance were decreased on leaves of *Parthenium hysterophorus* with *Epiblema strenuana* galls [13]. The Asian chestnut gall wasp was reported to reduce the photosynthesizing leaf area by around 40% when compared to a non-galled leaf. It also induces reduction in photosynthetic capacity (~60%) and stomatal conductance (~50%) [14]. It has also been noted that gall-inducing mites, such as *Vasates aceriscrumena,* may be the major drivers of age-dependent reductions in the physiological performance and growth of the canopy leaves of mature sugar maples (*Acer saccharum*) [15].

In contrast, the phyllodes of *Acacia pycnantha* with wasp-induced galls had higher photosynthetic rates as indicated by gas exchange than similarly aged control phyllodes without galls [10]. Photosynthesis (indicated by gas exchange), stomatal conductance, and water potential were increased on *Silphium integrifolium* leaves with *Antistrophus silphii* galls compared to ungalled shoots [16]. A scale insect on leaves of *Ilex aquifolium* also caused a higher PSΙΙ energy transduction efficiency, as indicated by chlorophyll fluorescence (Fv/Fm), in affected tissues relative to uninfected tissues [17].

The characterization of gall transcriptomes in grape leaves shows that galling insects increase their primary metabolic gene expression, including glycolysis, fermentation, and the transport of water, nutrients, and minerals in leaf-derived gall tissues, and decrease the expression of genes responsible for non-mevalonate and terpenoid synthesis, but increase the biosynthesis of shikimate and phenylpropanoid, which are secondary metabolites that alter the defense status of grapes [18]. Investigation of the metabolic responses of pteromalid wasp (*Trichilogaster acaciaelongifoliae*) larvae in bud galls on *Acacia longifolia* to reduced oxygen (O_2_) and elevated carbon dioxide (CO_2_) indicates that the larvae are tolerant to hypoxia/hypercarbia and are capable of reducing their respiratory rates to cope with hypercarbia [19]. Symbiosis between gall-inducing insects and fungi catalyze their expansion of resource use (niche expansion) and diversification (*i.e.*, the evolution of symbiotic interactions leads to niche expansion), which in turn catalyzes additional diversification [20].

In previous studies, we concluded that two leaf-derived cecidomyiid galls, the red ovoid galls induced by *D. taiwanensis* and the green obovate galls induced by *Daphnephila sueyenae*, are photoassimilative sinks in *Machilus thunbergii* (Lauraceae) leaves. This data also implies that insect-induced galls may have chlorophyll-deficient non-leaf green tissues composed to a very high extent of heterotrophic tissues and autotrophic tissues to a much lower extent [8,9,21].

The ‘Ambrosia’ gall midges, significant portion of the family Cecidomyiidae, are one of the most diverse and widespread groups of insects known to engage in symbiotic associations with fungi. The galls induced by these midges are typically lined internally with fungal hyphae, which the developing larva may feed upon [22]. The cup-shaped gall induced by *Bruggmanniella* sp. also contained an associated fungus. *Litsea acuminata* is an abundant and common subtropical tree species that is widely distributed in Taiwan. It is located 400 ~ 2,000 m above sea level (asl) in Taiwan, and can grow to 20 m in height with profuse branching. A cup-shaped gall induced by *Bruggmanniella* sp. on host leaves of *L. acuminata* was examined to investigate the relationship between this gall and its host leaves [23]. Our field observations revealed that *Bruggmanniella* larvae hatch from eggs in the spring, mine directly into leaf tissues, and remain undeveloped until fall. Galls then begin to develop around October and mature soon thereafter. The larvae develop into second and third instars within mature galls and emerge in early spring of the following year.

Little study has been done on the photosynthetic characteristics of gall midges and their relationship to the photosynthetic biochemical mechanisms of galls. In this study, we investigated the effects of galling by a midge on *L. acuminata* by measuring chlorophyll fluorescence, photosynthetic capacity, ultrastructural morphology, and biochemical composition of the gall and the host leaf.

## Results

### Photosynthetic pigments

Host plant leaves and their galls have different Car/Chl ratios in addition to great differences in Chl and Car content (Table 1). While galled or gall-free leaves contained around 2,000 and 1,000 μg/g DW of Chl and Car, respectively, and gall levels were reduced to 39 and 21 μg/g DW, respectively. That is, both the Chl and Car content of galls are only ~2% of the gall-free or galled leaves. While all the Chl a/b ratios of galls, and galled and gall-free leaves were the same (~2.7), the Car/Chl ratios were significantly different between galls and galled or gall-free leaves, the former being 0.54 and the latter 0.47.

**Table 1:** **Chlorophyll and carotenoid content in mature galls and two types of host leaves (n = 4)**

	**Chl a + b (μg/g DW)**	**Chl a/b**	**Car (μg/g DW)**	**Car/Chl**
**Gall-free leaves**	2138.8 ± 215.3^a^	2.68 ± 0.4^a^	1003.9 ± 78.0^a^	0.47 ± 0.4^b^
**Galled leaves**	1985.1 ± 262.2^a^	2.70 ± 0.4^a^	940.3 ± 120.0^a^	0.47 ± 0.3^b^
**Gall**	38.5 ± 3.8^b^	2.74 ± 0.1^a^	20.8 ± 1.4^b^	0.54 ± 0.2^a^

^a,b^ Significant difference (one-way ANOVA, Tukey’s honest significance difference test at p<0.05).

### Gas exchange capacity and Fv/Fm

**Figure 1:**
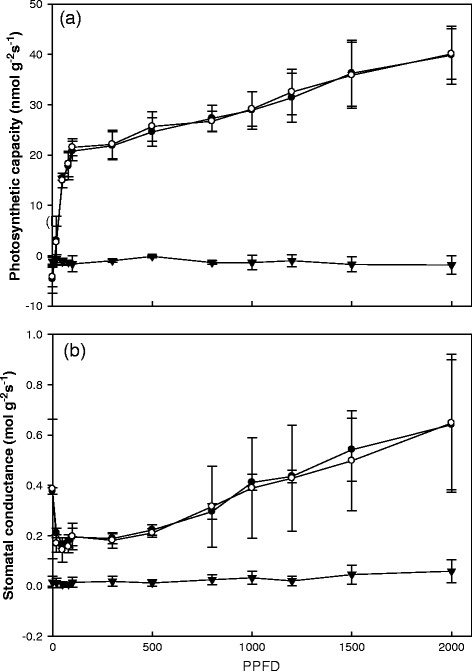
**Light response curves.** Light response curves for CO_2_ assimilation **(a)** and stomatal conductance **(b)** in gall (▼), gall-free (●), and galled *Litsea acuminate* (○), n = 4.

The light response curves for CO_2_ assimilation in galls and in the two types of leaves (*i.e.* galled and gall-free leaves) were determined. The photosynthetic light-saturation and light- saturation point of leaves of *L. acuminata* was about 100 μmol m^−2^ s^−1^ PPFD, respectively. No gas exchange was detected in any tested cup-shaped gall, whereas any type of host leaves exhibited a gas exchange level of 20 ~ 40 nmol g^−1^ s^−1^, as expected (Figure 1A). While stomatal conductance was not significantly different between galled and gall-free leaves, in cup-shaped galls it was always zero or close to it (Figure 1B).

### Exterior and interior gall surface

**Figure 2:**
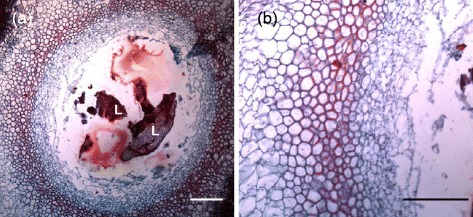
**Transverse sections of a cup-shaped gall. (a)** Gall chamber with larvae, 40×; **(b)** inside surface of gall, 100×; L: larvae. Scale bars: 500 μm.

**Figure 3:**
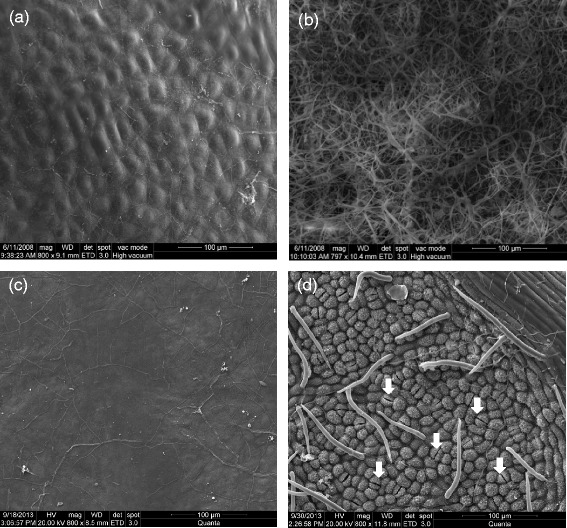
**Exterior and interior surface morphology of gall and host.** Exterior and interior surface morphology of a cup-shaped gall and host *Litsea acuminate*. **(a)** and **(b)** are exterior and interior surfaces of the gall, respectively; **(c)** and **(d)** show the adaxial and abaxial epidermis of a host *Litsea acuminate*. White arrows show guard cells.

Light microscopy revealed no stomata on the inside surfaces of gall chambers (Figure 2). Scanning electron microscopy of the exterior and interior surfaces of mature cup-shaped galls (Figure 3A and B) revealed no stomata on the outside surfaces of galls; however, their inside surfaces were covered with fungal hyphae (Figure 3B), there were no stomata insides gall tissues either. Trichome and trichome bases were found on exterior surfaces (Figure 3A and C). The stomata were observed on the abaxial epidermis but not on the adaxial epidermis of galled leaves (Figure 3C and D).

### The impact of photosynthetic rates on galls and host leaves

**Figure 4:**
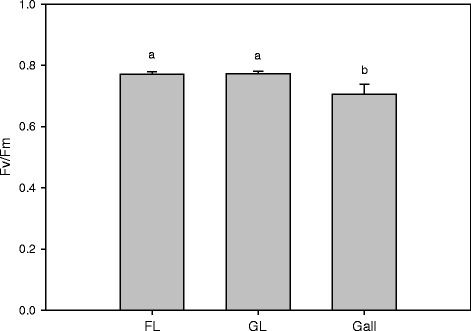
**Chlorophyll fluorescence**. The impacts of galling by a cecidomyiid on the chlorophyll fluorescence of gall-free (FL) and galled (GL) leaves and the gall of the host *Litsea acuminate*, n = 4.

Chlorophyll fluorescence was used to investigate whether the photosynthesis performance of galls and host leaves are different, as the former is cup-like and the latter is flat. No significant difference in Fv/Fm was observed between the two leaf types, indicating that the host leaves’ PSII efficiency might not be affected at all by the galling activity of the insect. The values of Fv/Fm were significant higher (10%) in the two leaf types than in galls (Figure 4).

### Confocal imagery of gall chloroplasts

**Figure 5:**
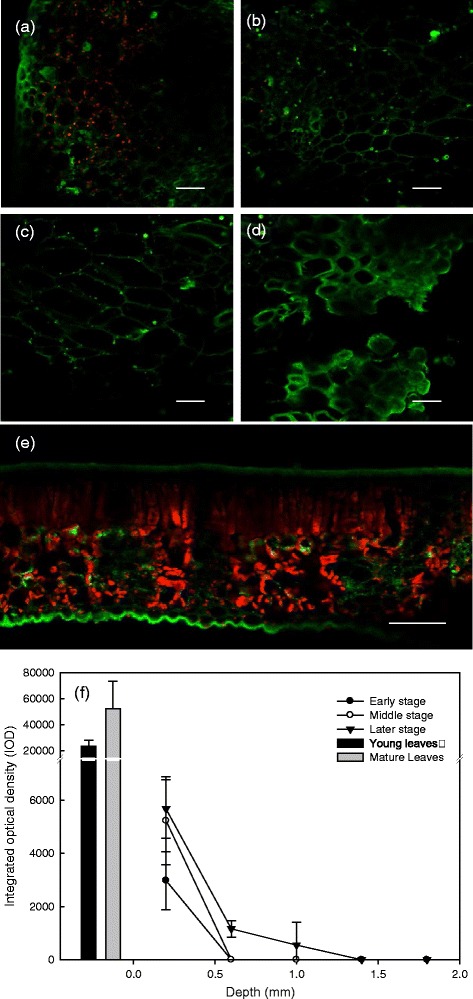
**Confocal images.** Confocal imagery of later-stage gall tissues from the outside to the inside of a larval chamber **(a ~ d)** and mature leaves **(e)**. The integrated optical density (IOD) of galls and gall-free and galled leaves **(f)** of the host *Litsea acuminata*. Data are the averages of thirty samples. Pink fluorescence shows the locations of chloroplasts. Error bars show SD. Scale bars: 100 μm.

Confocal laser scanning electron microscopy showed that while chloroplasts were relatively equally distributed in the mesophyll (*i.e.* palisade or spongy tissues), they were unequally distributed in insect-induced galls (Figure 5A-E). A gradient of chloroplast distribution existed in the galls, as indicated by confocal imagery and integrated optical density (IOD); *i.e.* outer gall tissues contain the highest density of chloroplasts and inner gall tissues have lower densities or none at all. Chloroplast fluorescence densities of young and mature host leaves were around 22,000 and 55,000 (IOD), respectively, whereas galls always have less than 5,800 (IOD), or none, regardless of whether galls were at early, middle, or later stages of growth (Figure 5F).

### Total soluble sugar, starch, free amino acid, and soluble protein

**Figure 6:**
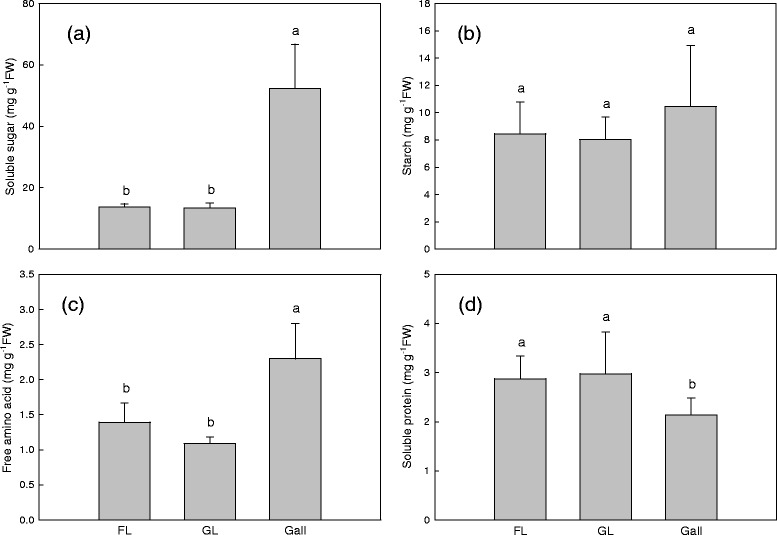
**Biochemical compositions.** Soluble sugar **(a)**, starch **(b)**, free amino acid **(c)**, and protein **(d)** contents of gall-free (FL) and galled (GL) *Litsea acuminata* leaves and galls, n = 8.

The total soluble sugar content in galls was three times higher than in the two types of host leaves (Figure 6A). However, starch contents showed no significant differences among galls and their two types of host leaves (Figure 6B). The two types of host leaves showed no significant differences in free amino acids and soluble proteins. However, insect-induced galls contained significantly higher levels of free amino acids, and significantly lower amounts of soluble proteins than the two types of host leaves (Figure 6C and D).

### Malondialdehyde (MDA*)*

**Figure 7:**
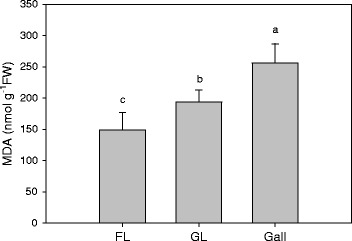
**Malonyl dialdehyde (MDA) levels.** (FL) gall-free and (GL) galled *Litsea acuminate* leaves and galls, n = 8.

The MDA contents of gall-free, and galled leaves and galls were around 150, 190, and 250 nmol g^−1^ FW, respectively. All three samples were significantly different from each other (Figure 7). The MDA content of gall was 31% higher than in galled leaves, and the latter were 26% higher than in gall-free leaves.

### Chloroplast ultrastructure

**Figure 8:**
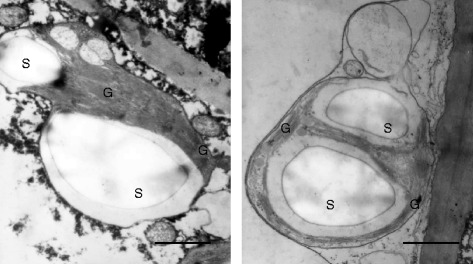
**Ultrastructural morphology of chloroplasts in galls.** Ultrastructural morphology of chloroplasts in galls on a host *Litsea acuminata* leaf. S, starch; G, grana. Scale bar: 1 μm.

Transmission electron microscopy showed that the chloroplasts of *L. acuminata* leaf galls possess a thylakoid morphology with normal stacked grana and are similar to those of other higher plants discussed in the literature (Figure 8). Besides normal grana stacking, mature cup-shaped galls have giant starch granules. These observations were ubiquitous during gall development from young to mature stages and from outer to inner layers (data not shown).

## Discussion

Previously, both red ovoid and green obovate galls on *Machilus thunbergii* (Lauraceae) leaves were found to be seriously deficient in the pigment-protein complexes of PSI and PSII throughout the entire period of gall formation, with a great decrease in carotenoids and chlorophyll-related compounds [8,9,24,25]. The large-scale deficiency in photosynthesis-related proteins and pigments (Table 1) results in incomplete light-harvesting by PSI and PSII on the thylakoid membrane of gall chloroplasts, which in turn indicates that the photosynthetic capacity must be much lower in galls than in host leaves, or even that no photosynthesis take place at all (26). These pigment-protein complexes contain either chlorophyll a or both chlorophyll a and b. Both types of pigment-protein complexes also possess several carotenoids that are thought to protect their associated polypeptides from photo-damage. Polypeptides in pigment-protein complexes are thought to orient chlorophyll molecules and perform their light harvesting energy transfer more quickly and efficiently [26].

Gall tissues typically have very low photosynthetic rates [12]. In the present study, no gas-exchange capacity was detected in gall tissues (Figure 1), which is probably due to the lack of stomata on exterior and interior gall surfaces (Figures 2 and 3). These results support the hypothesis that cup-shaped gall tissues serve as sinks of *L. acuminata* leaves as do the red ovoid and green obovate galls on *M. thunbergii* leaves [21].

Dorchin et al. [10] reported that wasp-induced galls may make a substantial contribution to carbon budgets, but gas-exchange in cup-shaped cecidomyiid galls was not detected either in this study or in many other kinds of galls [21]. These results demonstrate that physiological effects may differ among infections associated with different insect species and microenvironments. The significantly lower Fv/Fm values of galls relative to leaf surfaces indicates that galls have a much lower PSII energy transduction efficiency than non-infected leaf surface tissues (Figure 4). Furthermore, defects in the chlorophyll protein complexes of PSI and PSII in galls exhibited depressed efficiencies to the reaction centers of both photosystems [8,9,21]. Meanwhile, the stimulus of an insect on a host leaf results in a series of reactions in the host leaf, causing a shift from an autotrophic to a heterotrophic status in addition to gall development [21].

Koyama et al. [27] indicated that the improved performance of *Rhopalosiphum insertum* was ascribed to increased concentrations of amino acids in galled leaves. Gange and Nice [28] suggested that *Urophora cardui* gall inhabitants can manipulate N to an optimal level. Generally, gall tissues contain lower levels of N than ungalled plant tissues [26,29]. Lower plant N may have detrimental effects on insect herbivores, however, N is generally regarded as a crucial limiting nutrient for phytophagous arthropods, and it can increase the production of C-based secondary chemicals such as phenolics and may deter herbivore feeding [30]. These results suggest that any C-related metabolites in leaf tissues may be consumed to strengthen defense mechanisms against gall infections. Moreover, low levels of C and N may be an effect of the low levels of carotenoids and chlorophyll-related compounds in chlorophyll biosynthesis and degradation pathways. It could also be a result of low levels of photosynthesis-related proteins like RuBisCo in the stroma and light-harvesting protein complexes of the two photosystems on the thylakoid membrane and their corresponding mRNAs in galls [8,9,21,25,31]. It is known that RuBisCo and light-harvesting protein complexes comprise more than half of the stroma and thylakoid proteins in normal chloroplasts, respectively [26]. However, Hartley and Lawton [32] concluded that cynipid gall-formers may manipulate host N levels to their own advantage by preventing an increase in gall N levels in fertilized trees, and such an increase would reduce insect survival rates.

The efficiency of Fv/Fm in leaves was slightly affected by the galling activity (Figure 4) and the extent of its infection or gall number per leaves [33]. Retuerto et al. [17] reported that PSII (indicated by Fv/Fm) in galled leaves on holly trees was increased compared to gall-free leaves, and predicted that scale insects would increase photosynthesis. However, in our study, the values of Fv/Fm were not significantly different between galled and gall-free leaves, which agrees with the results of Aldea et al. [11] and Huang et al. [21].

A gradient of chloroplast distribution in terms of chloroplast number and fluorescence intensity was found in the cup-shaped galls of *L. acuminata* leaves (Figure 5), and in red ovoid and green obovate galls of *M. thunbergii* leaves (data not shown). Chloroplasts and fluorescence intensity were similar throughout mesophyll tissues. Chloroplasts were most abundant in outer gall tissues and least abundant (or absent) in inner gall tissues, which is further evidence that the highest photosynthetic capacity occurs in galls outer tissues (*i.e.* more autotrophic) and the lowest near the gall chamber (*i.e.* more heterotrophic).

Galls are reported to have significantly higher levels of soluble carbohydrates than leaves [34], and soluble sugar are higher in leaf blades of the European chestnut while galls are richer in starch [14]. In this study, gall tissues had much higher total soluble sugar levels than leaves (Figure 6), suggestive of a translocation of carbohydrates from leaves to galls, since the photosynthetic capacity of galls is much lower than that of the host leaves. Similar levels of starch in galls and host leaves can be caused either by high metabolic activity or by translocation of nutrients to galls [20,24]. The highest content of total soluble sugars in galls should indicate that the intake of carbohydrates is necessary as a nutrition source for galls. High concentrations of total soluble sugars in tissues of galls, together with the lower energy transduction efficiency of PSII, confirms that galls are sinks for host leaf photoassimilates [24,35]. Our present and previous results [20] support this hypothesis and agree with the findings of Bronner [3], Motta et al. [34], and Castro et al. [24].

The MDA content in the cup-shaped gall is around 31% higher than in the galled host leaf, which is around 26% higher than in the gall-free leaf (Figure 7). Our data strongly suggest that the gall-inducing insect significantly affected the lipid composition of the host leaf in plasma membrane level, and that the lipids of the cup-shaped gall are under higher oxidative stress than in the galled leaf, which in turn is under higher oxidative stress than in the gall-free leaf. The oxygen concentration in gall tissues is much lower than in galled or gall-free leaves [18], but gall tissues face higher oxidative stressing than host leaves. However, galls produce more secondary metabolites such as polyphenols, anthocyanins, flavanoids, and tannins [8,9] to strengthen the galls antioxidative capacity for counteracting higher oxidative stresses [36].

Cup-shaped galls contain giant starch granules and normal stacked grana (Figure 8). In this study, no stomata were found on exterior and interior gall surfaces nor was gas exchange detected on exterior gall surfaces. Therefore, it is possible that the photoassimilates of gall chloroplasts might be barely enough to support the galls, and the nutrition required by the larvae in the gall chamber might be translocated directly or indirectly from the host leaves. If so, CO_2_ and O_2_ cycling could be micro-environmentally self-balancing between mitochondria and chloroplasts in gall tissues. Since interior gall surfaces contain no effective stomata, it is perplexing how larvae in galls exchange CO_2_ and O_2_ over a period of more than ten months from oviposition to eclosion. One possibility is that gas exchange among gall, larvae, and fungi and for gall photosynthesis is via diffusion of CO_2_ and O_2_ through the gall’s innermost layer and the intercellular spaces of the inner tissue layers (Figure 2); that is, gas exchange can still take place via diffusion even though the internal surface is totally or partially covered by fungal hyphae [18].

Two kinds of food chains among larvae, galls, and leaves could exist in nature, depending on whether fungi occur inside galls. First, since abundant fungi grow inside the cup-shaped galls on *L. acuminata* leaves (Figure 3B) and the red ovoid and green galls on *M. thunbergii* leaves [20], it is possible that a micro-environmental food chain is generated as follows: host leaves → galls → fungi → larvae. The second possible food chain is host leaves → galls → larvae if the inside of the galls have no fungi and no stomata as in the ball-shaped or globular galls induced by *Trioza shuiliensis* (Yang) on *Machilus japonica* (data not shown). This question is currently under investigation by our group.

Many chlorophyll-deficient mutants have been reported in barley, pea, maize, wheat, sweet clover, rice, soybean, sugar beet, *Arabidopsis thaliana*, *Chlamydomonas,* variegated plants, and other plants. Except for several cases, all chlorophyll-deficient mutants reveal reductions in chlorophyll content, higher ratios of chlorophyll a/b, immature ultrastructures in the thylakoid membrane, marked changes in pigment-protein complexes, and a general sensitivity to temperature, light intensity, and photoperiod [37]. Mungbean testa [38] and insect-induced galls, both are deficient in pigment-protein complexes of PSI and PSII, can be recognized either as a chlorophyll-deficient mutation of the leaf or a non-leaf green tissue with abnormal morphology. The insect attack causes a transformation from a flat leaf to a cup-shaped gall with varying appearance and color. However, while chlorophyll a/b ratios of insect-induced galls and mungbean testa are below the average (2.5 ~ 3.0) for leaves, the ratios for chlorophyll-deficient mutants are between 4 and **∞** [37]. The incomplete organization of PSI and PSII may affect gall photosynthesis light-harvesting, energy transfer, and photochemical energy conversion performed in pigment-protein complexes.

## Conclusions

All the current evidence indicates that galling cecidomyiid *Bruggmanniella* sp. insects transform the photoassimilative abilities of *L. acuminata* and *M. thunbergii* leaves into gall sinks. This is strongly indicated in the present study because there is (1) no stomata in gall external and internal surfaces, (2) no gas exchange in the external surfaces of galls, and (3) a great increase in soluble sugars in galls. This conclusion is supported by other authors that have noted (1) a great reduction in carotenoids and chlorophyll-related compounds, (2) a deficiency in pigment-protein complexes and incomplete light-harvesting antennae in PSI and PSII supramolecules, (3) insect-induced cecidomyiid galls deficient in light-harvesting protein complex II showing normal grana stacking, and (4) a great reduction in secondary metabolites like anthocyanins and tannins [8,9,20,24,31,39]. In short, leaf-derived cecidomyiid galls are novel sinks in *L. acuminata* leaves. Insect-induced galls may be a new organ that is mostly heterotrophic but still retains an autotrophic function. The ratio between heterotrophy and autotrophy in galls requires further exploration.

## Methods

### Plants and galls

**Figure 9:**
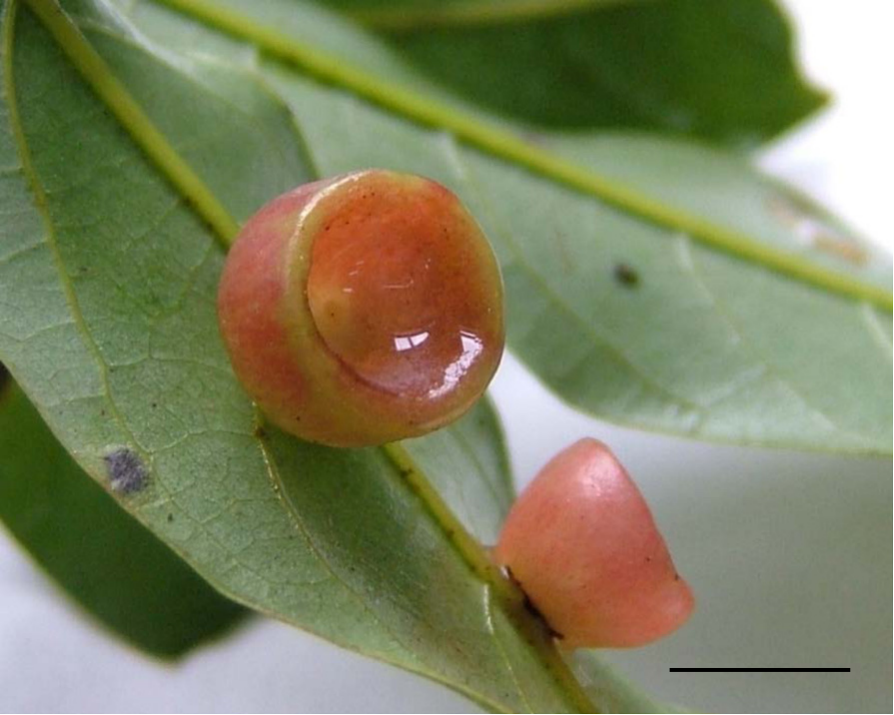
**Morphology of cup-shaped galls.** Morphology of cup-shaped galls induced by *Bruggmanniella sp.* on host leaves of *Litsea acuminata*. Galls reside on the lower epidermal midrib or vein of host leaves. Scale bars: 1 cm.

Cup-shaped galls (Figure 9) induced by an unidentified gall midge on *Litsea acuminata* were collected from 800 m asl in the Mt. Datun region, Yangmingshan National Park (approximately 11,455 hectares, 200 to 1,120 meters asl) located in the north-western part of Taipei, Taiwan. The gall usually takes about four months to develop from oviposition to maturity. The gall is pink or light-green and its center is concave, looking like a crystallite cup or bowl (Table 1). We sampled trees for cup-shaped galls and collected data from gall-infected and non-galled leaves in the early spring (February and March) of 2009–2011 when galls were mature. Tissues of cup-shaped galls, galled leaves (GL), and gall-free leaves (FL) were examined. Samples were kept on ice until sampling was finished. Galls were dissected to separate larvae from plant tissues and fixed for confocal imagery microscopy and scanning and transmission electron microscopy. Dissected galls were lyophilized, and stored at −20°C until analysis.

### Pigment analysis

Mature leaves and galls were frozen with liquid nitrogen and then extracted with 80% acetone. The concentrations of chlorophyll and carotenoids (Car) were determined according to a combined procedure described by Yang et al. [40].

### Photosynthetic capacity and chlorophyll fluorescence

Chlorophyll fluorescence was detected (for 20 min with leaf clips) at dawn on dark-adapted galls and uninfected tissues of the same leaf at room temperature with a Pocket Plant Efficiency Analyzer (Hansatech, U.K.). The maximum quantum efficiency of PSII (Fv/Fm) was calculated from (Fm-Fo)/Fm, where Fo, Fm, and Fv are ground fluorescence, maximum fluorescence of the dark-adapted subjects, and maximum variable fluorescence, respectively [41]. The light response curves of photosynthesis were measured with a portable, open-flow gas exchange system connected to a leaf chamber and LED light source (model 6400, LI-COR, CA, USA). Photosynthetic capacity was measured at saturating light intensities under 800 μmol m^−2^ s^−1^ photosynthetic photon flux density (PPFD) at ambient temperature. All measurements were taken before 11:00 a.m. to avoid the midday depression in photosynthesis. Fluorometer dark leaf clips were circular with diameters of 5.5 mm, which are considerably smaller than the typical gall diameter of 8–20 mm. Gas exchange was measured with a 6 cm^2^ cuvette. The area surrounding the gall was sealed with black tape to ensure that the measurement of gas exchange was solely from the gall and excluded the effects of ungalled leaf tissues. Chlorophyll fluorescence and photosynthetic rates of galls and host leaves (FL and GL) were then examined.

### Light microscopy

Galls and leaves of the host plant were cleaned and fixed in FAA solution (formalin, acetic acid, 50% ethanol; 1:1:18) for 24–48 h. Fixed samples were gradually dehydrated in an ethanol series, slowly infiltrated and embedded in paraffin, and then sectioned on a manual rotary microtome (American Optical 820, Leica, USA) using a razor blade. The 15 μm thick sections were examined using a light microscope (Leitz Laborlux S, Leitz, German).

### Scanning electron microscopy

Detached young and mature galls, and mature leaves were fixed for at least 3 h in 2.5% glutaraldehyde and then washed three times with 0.1 M phosphate buffer. Each wash took 15 min. A series of ethanol mixtures (30, 50, 75, 90, and 100%) were used to dehydrate fixed samples followed by 100% acetone. A thin gold film was set on the sample’s surface [42]. The morphology of the gall epidermis was examined with a Zeiss DSM 950 scanning electron microscope.

### Transmission electron microscopy

The inner parts of galls were collected, cut into small cubes, and placed in fixation solution containing 2.5% glutaraldehyde and 4% paraformaldehyde in 0.1 M sodium phosphate buffer (pH 7.0) in the field. The samples were washed three 20-min rinses, post-fixed in 1% osmium tetroxide for 2 h, dehydrated through an ethanol series, infiltrated and embedded in Spurr’s resin [43], and then polymerized at 70°C for 8 h. The ultrathin sections (70–90 nm) were collected and stained with ethanol uranyl acetate and lead citrate. Thylakoid morphology was observed with a Philips CM 100 transmission electron microscope at 75 kV.

### Confocal laser scanning electron microscopy

Hand-cut sections of leaves and galls were placed on slides in distilled water. Photographic images were captured with a Zeiss LSM510 Meta confocal microscope with a Plan-Apochromat 20×/0.6 objective. Specimens were excited by an 8% Argon 488 nm laser and emission signals were detected by a photon multiplier tube (Main beam splitter: HFT 488; beam splitter 1: Mirror; beam splitter 2: NFT 545; BP 650–710 IR, Zeiss, Germany). Chlorophyll red auto-fluorescence indicated chloroplast localization. Confocal images were analyzed by Image-Pro Plus software (Medica Cybernetics, USA). Color-cube-based segmentation was used to select only those shades of red that were in the area of interest. The integrated optical density (IOD) of red staining was measured and indicated a positive correlation to chlorophyll fluorescence intensity.

### Total soluble sugars and free amino acids

Samples (0.2 g fresh weight) were placed into a 15 mL tube and then 5 mL of distilled water was added and mixed in. The supernatant was collected after 30 min in a water bath at 85°C. This step was repeated once and then distilled water was added to obtain 10 mL of the collected extract. The soluble sugar content was then determined using the sulfuric acid anthrone method [44] at a wavelength of 630 nm and the free amino acid content was determined using the ninhydrin method [45] at a wavelength of 570 nm.

### Starch content

Starch was extracted according to the method of Takahashi et al. [46]. The residue obtained from distilled-water extraction was dried and added to 1 mL of distilled water. This mixture was placed in a water bath for 30 min at 100°C. The resulting gelatinized starch was digested after cooling with 1 mL 9.2 N perchloric acid for 10 min. Two milliliters of distilled water was added and the mixture was centrifuged at 8,000 *g* for 6 min. After the extract was transferred to a 15 mL tube, 1 mL of 4.6 N perchloric acid was added and stirred for 10 min. Three milliliters of distilled water were added to the final volume after centrifugation. Starch contents were determined using the same method as for soluble sugars.

### Soluble protein content

Total protein was measured using the method of Bradford [47]. Samples (0.2 g fresh weight) were ground up in mortar with liquid nitrogen, to which 3 mL of a phosphate buffered solution (pH 7.0) was added. The extract was centrifuged at 13,000 *g* for 15 min at 4°C and 0.1 mL of the supernatant was combined with 4.9 mL of Coomassie brilliant blue G-250 solution (0.1 g L^−1^). The soluble protein content was determined after 2 min at a wavelength of 595 nm.

### Lipid peroxidation

To measure the malondialhyde (MDA) content, fresh samples (0.2 g) were ground in a cold mortar containing 1.8 ml of 5% trichloroacetic acid. After centrifugation at 10,000 *g* for 5 min at 20°C, 1 ml of supernatant with 4 ml of 0.5% thiobarbituric acid reagent (0.5% [m/v] thiobarbituric acid dissolved in 20% [m/v] trichloroacetic acid) was mixed in, heated at 95°C for 30 min, and then quickly cooled and centrifuged at 2,000 *g* for 10 min. Absorbance was determined at 532 and 600 nm. The MDA content was computed using a standard curve relating MDA concentrations to absorbance [48].

### Statistical analysis

Differences in photosynthetic parameters among tissues were examined using a completely randomized analysis of variance. For significant values, means were separated by Tukey’s honest significance difference test at *p* < 0.05. Relationships between parameters were examined using simple linear regression models. All statistical analyses were conducted using JMP software, version 5.01 (SAS Institute, Cary, NC).

## Abbreviations

C: Carbon
N: Nitrogen
CO_2_: Carbon dioxide
O_2_: Oxygen
PS: Photosystem
Fo: Background fluorescence
Fm: Maximum dark-adapted fluorescence
Fv: Maximum variable fluorescence
GL: Galled leaves
FL: Gall-free leaves
MDA: Malondialdehyde
LED: Light emitting diode
PPFD: Photosynthetic photon flux density
